# TRPV4 plays a role in breast cancer cell migration via Ca^2+^-dependent activation of AKT and downregulation of E-cadherin cell cortex protein

**DOI:** 10.1038/oncsis.2017.39

**Published:** 2017-05-22

**Authors:** W H Lee, L Y Choong, T H Jin, N N Mon, S Chong, C S Liew, T Putti, S Y Lu, C Harteneck, Y P Lim

**Affiliations:** 1 Department of Biochemistry, Yong Loo Lin School of Medicine, National University of Singapore, Singapore, Singapore; 2 Department of Pathology, National University of Singapore and National University Hospital, Singapore, Singapore; 3 Department of Pharmacology and Experimental Therapy, Institute of Experimental and Clinical Pharmacology and Toxicology, Eberhard Karls University Hospitals and Clinics, Tübingen, Germany; 4 NUS Graduate School for Integrative Sciences and Engineering, Singapore, Singapore; 5 National University Cancer Institute, National University Health System, Singapore, Singapore

## Abstract

TRPV4 belongs to the ‘Transient Receptor Potential’ (TRP) superfamily. It has been identified to profoundly affect a variety of physiological processes, including nociception, heat sensation and inflammation. Unlike other TRP superfamily channels, its role in cancers are unknown until recently when we reported TRPV4 to be required for cancer cell softness that may promote breast cancer cell extravasation and metastasis. Here, we elucidated the molecular mechanisms mediated by TRPV4 in the metastatic breast cancer cells. TRPV4-mediated signaling was demonstrated to involve Ca^2+^-dependent activation of AKT and downregulation of E-cadherin expression, which was abolished upon TRPV4 silencing. Functionally, TRPV4-enhanced breast caner cell transendothelial migration requires AKT activity while a combination of transcriptional and post-translational regulation contributed to the TRPV4-mediated E-cadherin downregulation. Finally, mass spectrometry analysis revealed that TRPV4 is required for the expression of a network of secreted proteins involved in extracellular matrix remodeling. In conclusion, TRPV4 may regulate breast cancer metastasis by regulating cell softness through the Ca^2+^-dependent AKT-E-cadherin signaling axis and regulation of the expression of extracellular proteins.

## Introduction

Despite the relatively high survival rate of patients with early-stage breast cancers, the 5-year survival rate after diagnosis of stage-4 breast cancer, involving spread of tumor cells to other organs, is considerably low (22%).^1^ Metastatic breast cancer remains a major challenge since some patients cannot benefit from curative surgery and eventually become refractory to chemo and targeted therapeutics.^2, 3^ There are much unmet needs for therapeutic intervention at the metastatic stage. Identification of metastasis-associated genes and elucidation of the molecular etiology of metastasis are avenues to increase the pipeline of drugs.^4^ Cell migration and tumor vascularization are often accompanied by changes in ion channel expression and/or activity.^5, 6, 7, 8, 9, 10^ This may eventually lead to deregulation of the intracellular signaling. In particular, Ca^2+^ channels are of key importance since Ca^2+^ is major second messenger regulating a diversity of vital cellular processes such as proliferation, apoptosis, gene transcription, migration and angiogenesis. This may eventually lead to deregulation of the intracellular signaling. In particular, Ca^2+^ channels are of key importance since Ca^2+^ is major second messenger regulating a diversity of vital cellular processes such as proliferation, apoptosis, gene transcription, migration and angiogenesis.

The transient receptor potential (TRP) multigene superfamily encodes integral membrane proteins that function as ion channels.^11^ Members of this family are conserved in yeast, invertebrates and vertebrates. The TRP family is subdivided into seven subfamilies: TRPC (canonical), TRPV (vanilloid), TRPM (melastatin), TRPP (polycystin), TRPML (mucolipin), TRPA (ankyrin) and TRPN (NOMPC-like); the latter is found only in invertebrates and fish.^11^ Transient receptor potential vanilloid 4 (TRPV4) is modestly permeable to Ca^2+^ with a permeability ratio PCa/PNa between 1 and 10. Ca^2+^ influx is a key trigger and regulator of neurotransmitter release and muscle contraction. Moreover, remodeling of Ca^2+^ influx pathways was found to associate with cancer cell proliferation and migration.^9^ TRPV4 channelopathies are linked to skeletal dysplasias.^12^ TRPV4 has been implicated as a potential target in angiogenesis.^13, 14^ Activation of TRPV4 promoted the migration of breast cancer-derived endothelial cells but not endothelial cells derived from normal breast cells and TRPV4 silencing reduced arachidonic acid-stimulated migration of breast cancer-derived endothelial cells. Using the phosphoproteomics approach, our lab previously identified TRPV4 to play a role in breast cancer metastasis.^15^ Furthermore, meta-analysis of public databases through multifunctional online tools (http://co.bmc.lu.se/gobo,http://kmplot.com/analysis), *TRPV4* expression in breast cancer patients was shown to correlate with a more aggressive phenotype, a poorer overall and distant metastasis-free survival (DMFS) in a multivariate analyses.^15^

Clinically, breast cancers that defined as ‘triple negative’ are generally associated with a poor prognosis and a lack of long-term effective therapies.^16, 17, 18^ This is in part due to an absence of ErbB2, estrogen and progesterone receptor overexpression for targeted hormonal therapies.^17^ Basal-like and Claudin-low breast cancer subtypes are also associated with poor prognosis and are in great need for new effective therapies.^19^ Pharmacological targeting of calcium influx pathways in breast cancer, together with the conventional regime, may provide alternative and more effective therapeutic intervention for those who do not respond well to the hormonal therapies.^20^ One of the major potential advantages of regulators of calcium influx as novel drug targets for cancer is the clear ability to design pharmacological modulators of Ca^2+^.^20^

In our previous study, we reported that TRPV4 promoted cancer cell extravasation and metastasis by conferring cancer cell softness.^15^ However, the expression of TRPV4 proteins in clinical samples and the molecular mechanism through which TRPV4 mediate metastasis are unclear. Here, we showed that TRPV4 is overexpressed in breast cancer tissues and its expression correlates with poor outcomes. Molecularly, TRPV4 activation induced protein kinase B (PKB/AKT) phosphorylation and downregulation of cell cortex proteins E-cadherin expression. Moreover, TRPV4 is required for the secretion of proteins involved in remodeling of the extracellular matrix.

## Results

### Up-regulation of TRPV4 protein expression in the breast cancer

**Figure 1:**
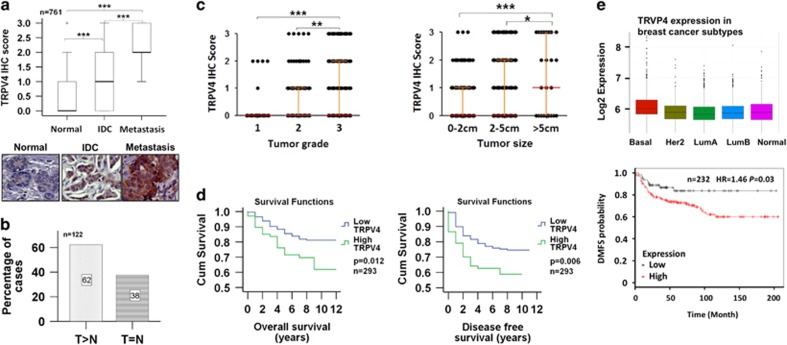
TRPV4 expression in clinical breast cancers. (**a**) Box plot of TRPV4 expression in normal, IDC and metastasis lesions. Statistical significance was calculated using Mann–Whitney *U* test. Representative images are shown at × 400 magnification. (**b**) Bar chart showing the percentages of cases of paired IDC tissues and adjacent normal breast tissues with the indicated trends. Statistical significance was calculated using Mann–Whitney *U* test (*n*=122). (**c**) Dot plots showing the correlation of TRPV4 expression with tumor grade and size. Statistical significance for the bar charts was calculated using Mann–Whitney *U* test. (**d**) Kaplan–Meier Analysis of TRPV4 expression with OS and disease-free survival. Statistical significance was estimated using log-rank test. (**e**) Clinical significance of TRPV4 overexpression. Upper: the box plot of TRPV4 gene expression across tumor subtypes using GENEANALYTICS. Lower: correlation of *TRPV4* expression with DMFS using KMplotter on a total of 232 basal subtype breast cancers.

To investigate whether TRPV4 has a role in clinical breast cancer, we performed immunohistochemistry (IHC) to examine the TRPV4 protein expression between the normal and the cancerous tissues. Analysis of 761 samples revealed that TRPV4 expression is significantly different between normal, invasive ductal carcinoma (IDC) and metastatic (Mets) lesions. The median TRPV4 IHC score increased from 0 in the normal tissues, to 1 and 2 in the IDC and Mets, respectively (Figure 1a). This indicates that cancer progression is accompanied by an increase in the TRPV4 expression, suggesting a possible link between TRPV4 overexpression and emergence of metastatic traits. Representative IHC images are shown in (Figure 1a). To examine more closely, we compared the expression of TRPV4 in 122 specimens comprising IDC and its adjacent normal tissues. Sixty-two percent (76/122) of the samples showed TRPV4 expression to be higher in IDC compared to normal tissues (Figure 1b). The specificity of the antibodies was confirmed by peptide competition studies using TRPV4-specific and control peptides (Supplementary Figure 1). Next, we inspect if TRPV4 expression correlates with key clinical-pathological parameters such as tumor grade and tumor size. The distribution plots show that, TRPV4 expression is significantly and positively correlated with grade and tumor size (Figure 1c).

Finally, the overall survival (OS) and disease-free survival (DFS) of patients (*n*=293) segregated into high (IHC score >1) versus low TRPV4 expression was analyzed (Figure 1d). Patients with high TRPV4 expression exhibited significantly poorer OS (*P*=0.012) and DFS (*P*=0.006) as compared to the TRPV4-low patients. The hazard ratio for OS and DFS are 1.935 (*P*=0.015) and 1.862 (*P*=0.008), respectively. TRPV4 expression with respect to DMFS and breast cancer subtype were also analyzed using KMplotter (http://kmplot.com/analysis/) and GeneAnalytics (http://geneanalytics.duhs.duke.edu/index.html), respectively. The former incorporated the gene expression data and survival information from GEO (Affymetrix microarrays only), EGA and TCGA while the latter used a 50-gene qPCR assay (PAM50) to identify the intrinsic biological subtypes using RNA isolated from formalin-fixed, paraffin-embedded tissue. The data below shows that TRPV4 is not only associated with more aggressive subtypes of breast cancer (basal subtype in particular) but also with poorer DMSF in the breast cancer patients with basal subtype (Figure 1e).

Taken together, TRPV4 overexpression is associated with poorer survival rate and cancer aggression. This is consistent with our previous data that implicated a mechanical role of TRPV4 overexpression in metastasis by promoting cancer cell softness and migration.^15^

### Activation of TRPV4 leads to phosphorylation of AKT, focal adhesion kinase (FAK) and downregulation of E-cadherin and β-catenin proteins

**Figure 2:**
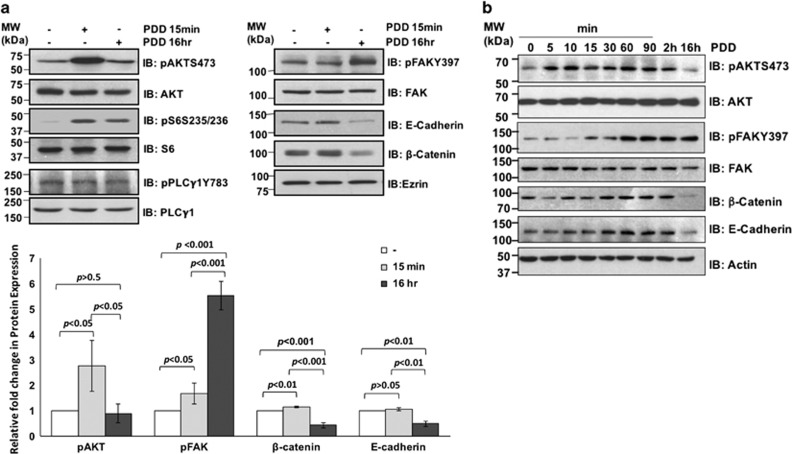
Signaling events following PDD-induced TRPV4 activation. (**a** and **b**) 4T07 cells were untreated or treated with 4α-PDD at 10 μm for the indicated time points before subjecting the cell lysates to immunoblotting with the indicated antibodies. The protein bands in **a** were measured and quantified using Image-J software.

The underlying mechanism on how TRPV4 contributes to the migratory/invasive phenotype is unclear. Since AKT and FAK signaling pathways are commonly associated with cell motility, we hypothesized that TRPV4’s function in cancer cell migration involve AKT and/or FAK-mediated signaling pathways. Treatment of 4T07 cells with 4α-phorbol 12,13-didecanoate (4α-PDD), a TRPV4-selective activator, for 15 min resulted in a significant increase in the phosphorylation/activation of AKT and its downstream substrate S6 but not their expressions (Figure 2a, left). Increase in the phosphorylation of FAK was observed marginally at 15 mins and increased at 16 h while downregulation of E-cadherin and β-catenin expression were observed at 16 h post stimulation (Figure 2a, right). Although phospholipase C (PLC) has been implicated in cell migration, it does not seem to mediate TRPV4 function since phosphorylation of PLC-γ1 was not induced following 4α-PDD treatment. This is consistent with the notion that 4α-PDD does not turn on the classical PKCs,^21^ which are activated by diacyl glycerol (DAG) following hydrolysis of PIP_2_ by PLC-γ1. A finer time course study showed that phosphorylation of AKT peaked at 10 min before weakening at 2 h and returned to basal level by 16 h of 4α-PDD treatment. FAK phosphorylation had a later onset peaking at 1 h but remain sustained until 16 h (Figure 2b). The expression of E-cadherin and β-catenin increased slightly after 30 min, sustained for 90 more min before decreasing at 16 h. A closer examination revealed that E-cadherin and β-catenin expression dropped progressively from 4, 8 and 16 h post 4α-PDD treatment (Figure 2b). Although maximum FAK activation occurred around 1 h, downregulation of E-cadherin and β-catenin occurred later.

### TRPV4 silencing abolished 4α-PDD-mediated signaling pathways

**Figure 3:**
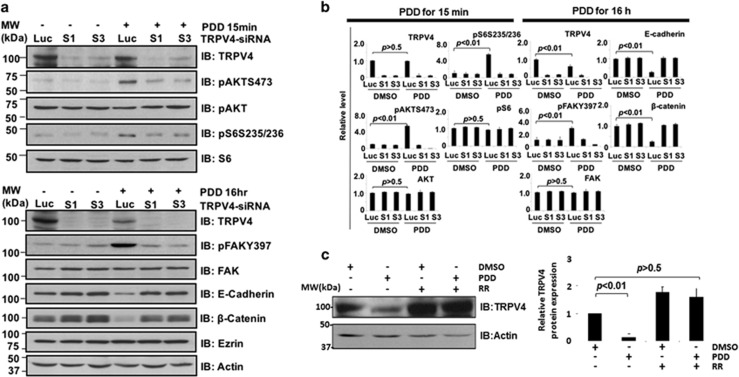
TRPV4-specific activation of downstream signaling pathways. (**a**) Control and *Trpv4* knocked-down cells were treated with DMSO or 4α-PDD and the lysates immunoblotted with the indicated antibodies. (**b**) The protein bands were measured and quantified using Image-J software. (**c**) 4T07 cells were either pre-treated or not with 10 μm ruthenium red for 1 h before stimulation with DMSO control or 4α-PDD. Lysates were then probed for TRPV4 and Actin control.

To prove that TRPV4 mediated the above observations, we repeated the 4α-PDD treatment in control versus TRPV4 knocked-down 4T07 cells. As shown in Figure 3a, the phosphorylation of AKT, S6 and FAK as well as the downregulation of E-cadherin and β-catenin expression were TRPV4 specific. Knockdown of *Trpv4* in the absence of 4α-PDD also resulted in a reproducible increase in E-cadherin and β-catenin expression, further supporting the notion that TRPV4 down-regulates these cytoskeleton-associated proteins. The bar charts showing the standard errors for all the immunoblots are shown in Figure 3b. Interestingly, TRPV4 was itself downregulated after 4α-PDD treatment for 16 h. Treatment of 4T07 cells with ruthenium red, a TRPV4 inhibitor, blocked the 4α-PDD-induced downregulation of TRPV4 (Figure 3c) implying that 4α-PDD-induced downregulation of TRPV4 is a reversible and regulated process. We also performed a similar experiment with 67NR (Supplementary Figure 2), which has low endogenous level of TRPV4. Upon TRPV4 activation by PDD, up-regulation of phospho-FAK and downregulation of E-cadherin were observed in TRPV4-high 4T07 compared to TRPV4-low 67NR cell line. Phosphorylation of AKT was also higher in 4T07 cells compared to 67RN although not as drastic as FAK. The reason for this observation is unclear. It is conceivable that the AKT pathway is sensitive to Ca^2+^ influx such that activation of the low endogenous level of TRPV4 in 67NR was sufficient to activate AKT.

### Signal transduction and function of TRPV4 were associated with increased intracellular Ca^2+^ concentration

**Figure 4:**
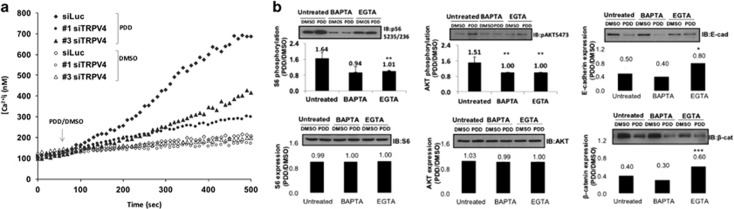
PDD-induced TRPV4 activation triggered Ca^2+^ influx. (**a**) Intracellular Ca^2+^ measurement in control or *Trpv4* knocked-down 4T07 cells treated with 10 μm of 4α-PDD or DMSO. *Luc*-DMSO=cells transfected with *Luc* control siRNA and treated with DMSO; *Luc*-PDD, cells transfected with *Luc* control siRNA and treated with 4α-PDD; S1-DMSO, cells transfected with *Trpv4* siRNA (sequence 1) and treated with DMSO; S1-PDD, cells transfected with *Trpv4* siRNA (sequence 1) and treated with 4α-PDD; S3-DMSO, cells transfected with *Trpv4*siRNA (sequence 3) and treated with DMSO; S3-PDD, cells transfected with TRPV4 siRNA (sequence 3) and treated with 4α-PDD. (**b**) Effects of chelating intracellular (by BAPTA-AM) and extracellular (by EGTA) Ca^2+^ on 4α-PDD-induced, TRPV4-mediated signal transduction. 4T07 cells were untreated or pre-treated with 20 μm of BAPTA-AM or 2 mm of EGTA for 1 h before being stimulated with 4α-PDD for 15 min (to assay for AKT activation) or 16 h (to assay for FAK activation and effects on E-cadherin and β-catenin). Cell lysates were then subject to immunoblotting using the indicated antibodies and bands densitometry performed using the ‘Image J’ software. The fold induction (ratio) was then obtained by dividing the values at PDD 15 min or PDD16 h over DMSO. Data points represent mean±s.e.m. of three independent experiments, *P<0.05, **P<0.01, and ***P<0.001*.*

Since TRPV4 is a Ca^2+^ permeable channel, we asked whether the above functions of TRPV4 were associated with its ability to regulate intracellular calcium, [Ca^2+^]_i_. Figure 4a shows that stimulation of control, but not *Trpv4* knocked-down cells, with 4α-PDD produced an increase in [Ca^2+^]_i_ indicating that TRPV4 was solely responsible for 4α-PDD-induced Ca^2+^ influx. Extracellular Ca^2+^ contributed solely to the increase in [Ca^2+^]_i_ following 4α-PDD treatment since ethylene glycol tetraacetic acid (EGTA) pretreatment completely abolished 4α-PDD-induced rise in [Ca^2+^]_i_ (data not shown). This confirms that the biochemical and function role of TRPV4 was associated with its ability to mediate Ca^2+^ influx.

The above observations revealed that TRPV4 was the major factor for PDD-induced Ca^2+^ influx. Based on this premise, we postulated that pretreatment of cells with intracellular and extracellular calcium chelators will diminish the effects of 4α-PDD-induced TRPV4 biochemical actions. In the absence of Ca^2+^ chelators, the fold phosphorylation of AKT and S6 in 4α-PDD-treated cells compared to DMSO control cells was 1.51 and 1.64 respectively (Figure 4b). Depletion of intra- and extra-cellular Ca^2+^ by BAPTA-AM or EGTA, respectively, significantly and drastically diminished 4α-PDD-induced autophosphorylation of AKT and phosphorylation of S6 as reflected by ratios of close to 1 in these conditions. The expression level of AKT and S6 was not affected by the presence of Ca^2+^ chelators (Figure 4b). Although the 4α-PDD-induced phosphorylation of FAK in the presence of Ca^2+^ chelators was significantly lower than no chelation (*P*<0.05), the effect was only modest (control: 1.7, BAPTA-AM: 1.4 and EGTA: 1.5, data not shown). This indicates that the early and acute AKT signaling cascade was dependent on TRPV4-mediated Ca^2+^ influx while the late FAK pathway was less so. Although 4α-PDD-induced downregulation of E-cadherin and β-catenin expression in the presence of EGTA was blocked considerably, no apparent difference was obtained with BAPTA-AM. The reason is not clear but it is evident that manipulation of Ca^2+^ has impact on TRPV4 downstream signaling events.

### AKT plays a role in TRPV4-mediated downregulation of E-cadherin and transendothelial migration

**Figure 5:**
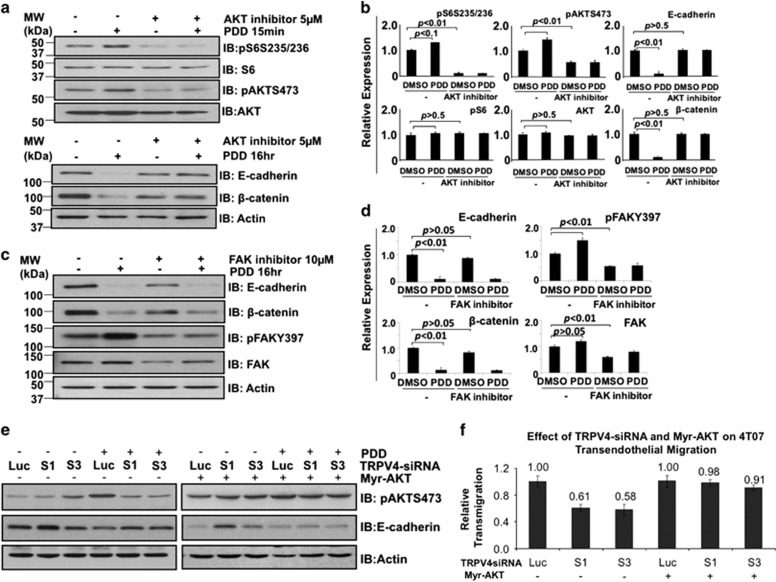
Investigating AKT and FAK as potential mediators of TRPV4-mediated downregulation of E-cadherin and β-catenin expression. Cells were pre-treated or not with 5 μm of AKT inhibitor IV (**a** and **b**) or 10 μm of FAK inhibitor (**c** and **d**) for 1 h before stimulation with 10 μm 4α-PDD for 15 min or 16 h as shown. Lysates were then probed with the indicated antibodies. Actin was used as a loading control. The levels of each protein shown in the bar charts were expressed using Actin as the denominator. (**e**) Effects of constitutively active AKT on *Trpv4* siRNA-mediated downregulation of E-cadherin expression. (**f**) 4T07 cells transfected with *Luc* or *Trpv4* siRNAs were overexpressed with or without Myr-AKT. 48 h post-transfection, cells were subject to transendothelial migration assay over a time course of 8 h duration. Data points represent mean±s.e.m. of three independent experiments.

As activation of AKT and FAK preceded downregulation of E-cadherin and β-catenin following 4α-PDD treatment, we asked whether AKT and/or FAK-mediated TRPV4-induced downregulation of these proteins. As we failed to knockdown AKT expression despite trying various small interfering RNA (siRNA) sequences, we resorted to pharmacological inhibitors. Figure 5a shows that the AKT inhibitor IV was effective in blocking 4α-PDD-induced phosphorylation of AKT and phosphorylation of S6. The bar charts showing the standard errors for the immunoblots are shown in Figure 5b. In addition, the AKT inhibitor could prevent 4α-PDD-induced downregulation of E-cadherin and β-catenin. In contrast, inhibition of FAK had no effect on PDD-induced and TRPV4-mediated downregulation of E-cadherin and β-catenin proteins (Figures 5c and d).

To confirm the role of AKT in TRPV4 signaling, we examined the effect of constitutively active AKT on E-cadherin expression before and after TRPV4 silencing. Exogenous expression of Myr-AKT was confirmed by anti-HA and anti-AKT blots (Figure 5e). As shown, constitutively active AKT could rescue the effects of TRPV4 knockdown on 4α-PDD-induced downregulation of E-cadherin expression (Figure 5e). In fact, constitutively active AKT alone reduced the expression of E-cadherin even in the absence of 4α-PDD. We did not observe clear changes with β-catenin in a separate experiment (data not shown). We also examined the role of constitutively active AKT for its potential in rescuing the effects of *Trpv4* knockdown on 4T07 transendothelial migration. Although TRPV4-silenced cells displayed lesser transendothelial migration compared to non-TRPV4 silenced cells, constitutively active AKT restored the transendothelial migration potential in 4T07 cells deprived of TRPV4 (Figure 5f). We further investigated whether AKT is required for TRPV4-mediated migration. 4T07 cells were treated with AKT inhibitor (5 μm) and/or arachidonic acid which (AA, 20 μm) in serum-free media over 24 h of time course before analysis. As for co-treatment, cells were first pre-treated with AKT inhibitor for 1 h. Arachidonic acid is used to activate TRPV4 channel as it is a well-known TRPV4 agonist.^14^ In fact, others have demonstrated tumor-derived endothelial cell migration mediated by TRPV4 via arachidonic acid-activated actin remodeling.^14^ The results demonstrated that AKT is required for TRPV4-mediated wound healing and transendothelial migration (Supplementary Figure 3). Western blots show that AA (20 μm) activated AKT while AKT inhibitor (5 μm) effectively blocked AKT activation. Collectively, the data support the role of AKT as a mediator of TRPV4 signaling and transendothelial migration of breast cancer cells.

### TRPV4 mediated E-cadherin downregulation by a combination of transcriptional and post-translational regulation

**Figure 6:**
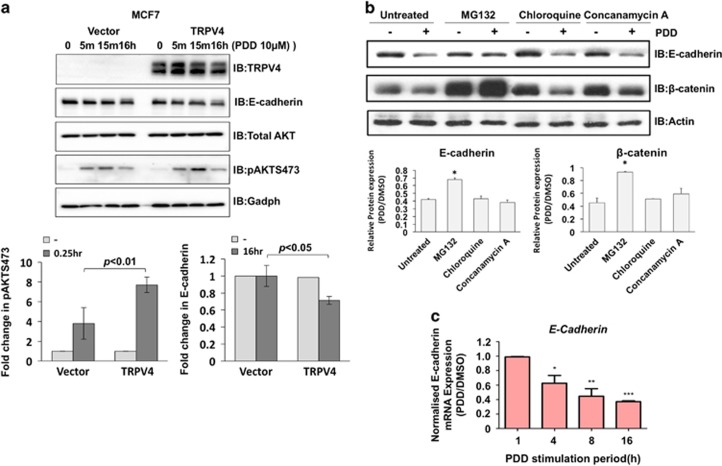
TRPV4 activation induced E-cadherin downregulation through a combination of transcriptional and post-translational mechanism. (**a**) MCF7 cell line was transiently transduced with vector control or TRPV4-expressing retrovirus. Cells were treated with PDD at indicated time points. Cell lysates were then Western blotted with the indicated antibodies (top panel). Following densitometry, relative expression of pAKT and E-cadherin were normalized with total AKT and Gadph, respectively, and plotted as bar charts (bottom panels). (**b**) Cells were pre-treated for 1 h with proteasomal inhibitor MG132 (10 μm), lysosomal inhibitors chloroquine (100 μm) or concanamycin A (100 nm) before treatment with 4α-PDD or DMSO. Lysates were then immunoblotted with the indicated antibodies. Bar charts are the densitometry of pAKT and E-cadherin protein expression levels. Relative expression of pAKT and E-cahderin were normalized with total AKT and Gadph, respectively. Error bars show mean±s.e.m. (*n*=3). (**c**) The mRNA expression of *E-cadherin* in 4T07 cells upon different durations of 4α-PDD stimulation was examined using real-time PCR. Error bars show mean±s.e.m.

To examine whether TRPV4 also has effects on the AKT and E-cadherin expression in human breast cell line, MCF7 cell line was transiently transduced by either vector control or TRPV4-expressing retrovirus. At 48 h of retroviral transduction, cells were treated with PDD at various time points as indicated in Figure 6a. In line with the 4T07 cells, MCF7 cells transiently transduced with TRPV4-expressed retroviruses showed a higher activation of AKT at 15 min post-PDD treatment. The higher activation of AKT was not due to increased expression as changes in total AKT expression levels in TRPV4-overexpressing cells were not observed. The increase in pAKT is concomitant with a decrease in E-cadherin expression compared to the control after 16 h of PDD treatment (Figure 6a). Similar observations were also obtained in HeLa cells (Supplementary Figure 4). Therefore, TRPV4-mediated reduced expression in E-cadherin could be recapitulated in the human context. To investigate whether downregulation of E-cadherin and β-catenin expression following 4α-PDD treatment were caused by protein turnover, proteasomal inhibitors (MG132) and lysosomal inhibitors (chloroquine and concanamycin A) were used to pre-treat 4T07 cells before stimulation with 4α-PDD. Figure 6b revealed that MG132 but not the lysosomal inhibitors significantly blocked 4α-PDD-induced decrease in E-cadherin and β-catenin expression. However, the blockage of E-cadherin by MG132 was not complete. Since E-cadherin is also regulated by transcriptional repression and epigenetics,^22, 23^ we investigated whether *E-cadherin* mRNA levels were altered upon TRPV4 activation. Quantitative real-time PCR revealed that *E-cadherin* mRNA reduced progressive and significantly 4 h after 4α-PDD treatment (Figure 6c). Collectively, the data suggest that TRPV4-mediated E-cadherin downregulation was achieved effectively via a combination of transcriptional and post-translational regulation.

### TRPV4 regulates a network of proteins involved in cytoskeleton and extracellular matrix remodeling

Besides AKT, FAK, E-cadherin and β-catenin, other targets are likely to participate in TRPV4-mediated metastasis. In addition, AKT has also been shown to contribute to tumor invasion and metastasis by promoting the secretion of tumor-associated proteins.^24, 25^ Since secreted proteins are both critical mediators of metastasis and attractive drug targets, we employed ITRAQ-based mass spectrometry to profile the levels of secreted proteins that are specifically upregulated by TRPV4 upon 4α-PDD treatment. Control or TRPV4-specific siRNA transfected 4T07 cells were treated with either DMSO or 4α-PDD for 16 h. Conditioned media was harvested, iTRAQ labeled and analyzed by mass spectrometry as per our previous study.^26^

**Figure 7:**
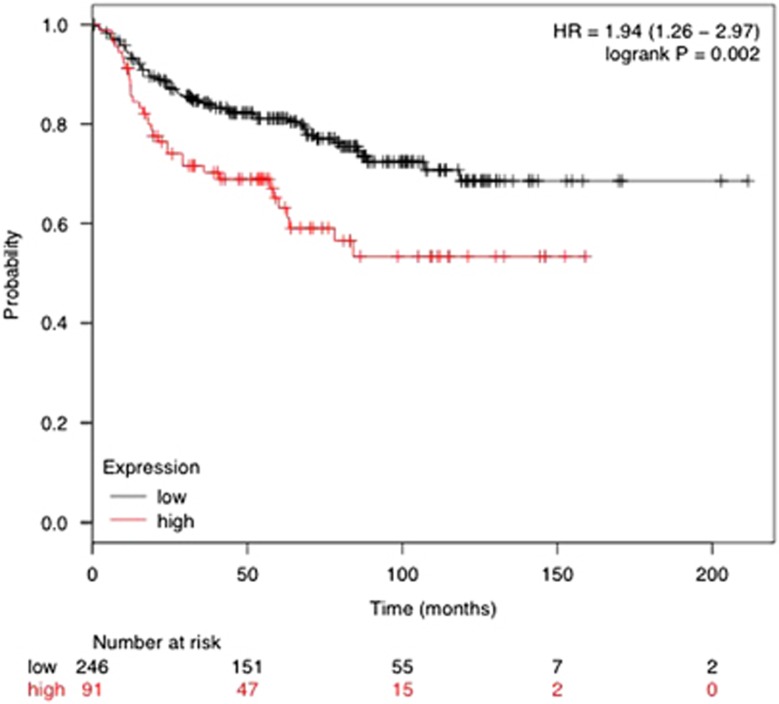
Correlation of *TALIN* expression in human breast cancers (*n*=1946) segregated by their lymph node status with distant metastasis-free survival (DMFS).

In total, 540 proteins were detected with 95% confidence (data not shown) while 128 proteins were found to have differences in expression levels in one or more conditions (Supplementary Table 1). Of interest are proteins whose expressions were affected by 4α-PDD compared to DMSO treatment in control siRNA transfected cells but not in *Trpv4* knockeddown cells as these represent specific targets of TRPV4. There were 27 such proteins of which 17 were authentic classical secreted proteins with signal peptide (*n*=9) or found in exosomes (*n*=8), whereas the mode of secretion for the rest remained unconfirmed (Table 1). Using Metacore analysis, the top 3 functional networks most significantly associated with the 27 proteins were cytoskeletal rearrangement, extracellular matrix remodeling and cell adhesion (Table 2). The specific roles of the 5 genes, *Fn1*, *Clu*, *Tubb2c*, *Tln1* and *Spp1* identified to be involved in the above-mentioned process networks, with respect to TRPV4 functions remained to be investigated but genes such as *Talin* (*Tln1*, upregulated by TRPV4) is well known to promote metastasis.^27^ Other genes upregulated by TRPV4, for example, *Transgelin*, *Trim28* and *Hdgf* are also involved in cancer progression^28, 29, 30, 31, 32, 33, 34, 35^ and could also potentially mediate metastasis following exosomes uptake by neighboring cells. Talin is an interesting target for further study as it exhibits the greatest relative change in its expression (Table 1). One can not exclude the possibility that at the physiological level, changes in the TRPV4 basal expression alone in the absence of agonist may also have an impact on its downstream signaling events. We thus further examine the data set for proteins whose expression was affected by TRPV4 KD alone (Table 3). Only the candidates which were consistently being upregulated or downregulated by S1 and S3 TRPV4-specific siRNAs with statistical significance (*P*<0.05) were selected. CDK5 emerges as an interesting candidate since its activity has been recently linked to the metastatic process in some cancers,^36, 37, 38, 39^ possibility by reducing the activity of the actin regulator protein, caldesmon. To further examine the significance of Talin to clinical human breast cancers, we resorted to meta-analysis of public databases through a multifunctional online tool, Kaplan–Meier plotter (KMplotter) that allows different analyses to be performed in a 4142-sample breast tumor data set with a mean follow-up of 69 months. KMplotter integrates the database from GEO (Affymetrix microarrays only), EGA and TCGA. High level of Talin was found to be associated with poorer DMFS in the patients with positive lymph node status (Figure 7). In contrast, DMFS rate in the patients with negative lymph node status was independent on Talin expression levels (data not shown). The data indicates relevance of Talin expression levels with metastatic breast cancer and supports the notion that TRPV4 and Talin may cooperate to promote cancer metastasis. Future studies shall clarify this hypothesis.

## Discussion

The TRP proteins are one of the major classes of ion channels on the cell surface (the voltage-gated channels being another one) that facilitates influx of Ca^2+^ from extracellular surface into the cytosol. Owing to their roles in cation flux including calcium homeostasis, it is not surprising that some members of the TRP superfamily have been implicated in human diseases and cancer. Dysregulation of the expression of TRPM1, TRPM8, TRPC6, TRPV1, TRPV2 and TRPV6 have been observed in prostate, ovarian, breast, liver and bladder cancers.^40, 41, 42^ TRPV4 has not been reported to play roles in cancer although it would have been inadvertently observed to be elevated in cancer from various DNA microarray studies.

There are contradictory reports on the role of TRPV4 in cell mobility. While Zaninetti *et al.*^43^ showed that TRPV4 activation reduced migration of neuroendocrine cells, Martin *et al.* and Fiorio *et al.* found TRPV4 to be capable of mediating migration of pulmonary artery smooth muscle and AA-induced migration of endothelial cells, respectively.^14, 44^ In this study, we showed that TRPV4-mediated AKT pathway is crucial for transendothelial migration of breast cancer cells. This is accompanied by downregulation of E-cadherin & β-catenin expression. Our results concur with the observation that squamous cell carcinoma lines engineered to express constitutively active AKT resulted in the downregulation of the epithelial markers such as E-cadherin.^45^ Moreover, Ca^2+^-dependent phosphorylation of AKT following activation of TRPV4 by 4α-PDD is consistent with the report by Danciu *et al.*^46^ The group showed that osteoblasts that were uniaxially stretched led to a significant increase in intracellular Ca^2+^ concentrations resulting in the phosphorylation of PI3K, AKT and its downstream transcription factor targets, FOXO1 and AFX.^46^

Based on the time course results (Figure 2b), FAK is activated/phosphorylated maximally at 1 h post 4α-PDD and sustained up to 16 h although TRPV4 was downregulated at 16 h. There is some resemblance between TRPV4 and the receptor tyrosine kinases in that they are modulated by E3 ligases following receptor activation.^47^ After signals are transduced from the ligand to the receptor, the latter undergoes modulation while downstream signals propagate on. This could explain the sustained activation of FAK while TRPV4 becomes downregulated. The mechanism of how FAK activation was sustained in this study is not clear. Study by *Thodeti et al.*^48^ showed that PI3K/AKT, activated by TRPV4-mediated calcium influx, is upstream of β1 integrin activation in the mechanically stretched endothelial cells. FAK binds to the cytoplasmic domain of β1 integrin, resulting in autophosphorylation of FAK at Y397.^49^ Interestingly, FAK activation appears to provide a positive feedback loop. *Garcia et al.*^49^ reported that FAK activity can enhance integrin activation. This may explain the sustained expression of pFAK^Y397^ by PDD at 16 h but further confirmatory studies are required.

Functional and physical links between TRPV4 and the cytoskeletal components were found to be critical to the cellular processes as the interactions between them not only modulate cytoskeletal dynamics and also TRPV4 channel activity.^50^ The formation of TRPV4 in a supra-molecular complex containing cytoskeletal proteins and regulator kinases hence is important to the mechanotransduction in the cells. Moreover, E-cadherin and β-catenin transmembrane proteins as part of the core of the adherens junction are essential for the maintenance of architecture and integrity of epithelial tissues.^51^ The intracellular domain of the cadherin molecule interacts with proteins of cytoplasmic plaque in catenins that link it to actin filaments to maintain the stability of adherens junctions. TRPV4 has been reported to interact with β-catenin and the TRPV4/ β- catenin complex can interact with E-cadherin.^52^ Our observation that TRPV4 regulates the stability of E-cadherin/ β-catenin is therefore not surprising although in-depth studies would be needed to identify the specific regulator of E-cadherin expression in TRPV4 signaling. Our data suggested a combination of transcriptional and post-translational regulation leading to TRPV4-mediated E-cadherin downregulation. It is possible that transcriptional repressors like Twist/Snail/Slug and E3 ligases such as Hakai are potential TRPV4 regulators.

After mapping AKT/E-cad signaling axis as a potential mode of action of TRPV4 in metastasis, secretomics was performed to attain a more comprehensive of how TRPV4 regulates metastasis by modulating the tumor microenvironment. Our secretomics data revealed that Talin expression, amongst all the candidates, was most drastically changed (by twofold increase) when TRPV4 was activated by 4α-PDD. Talin, a focal adhesion complex protein that regulates integrin interactions with ECM, has been shown to play a key role in a wide variety of integrin-mediated events both *in vitro* and *in vivo*. Many studies suggested its role in tumorigenesis, migration and metastasis.^53, 54, 55^ Moreover, high levels of Talin in the blood serum has been detected in the patients with colon and liver.^56, 57^ In a recent study by Sakamoto *et al.*^55^, Talin was found to engage in focal adhesion interactions with the AKT signaling as the intracellular survival mechanisms to confer anoikis resistance and promote prostate cancer cell invasion. The link between TRPV4 and Talin is novel and AKT could well be a mediator between them. Another interesting observation was the positive correlation between TRPV4 expression levels and the downstream secretion of CDK5. CDK5, kinase protein, may be transferred between cancer cells or be secreted to modulate tumor microenvironment and angiogenesis by tumor-associated exosomes. CDK5 has been well established as centrally involved in regulating migration of neuronal progenitor cells during brain development in embryogenesis,^39, 58, 59^ whereas extraneuronal reactivation of CDK5 in malignant tumors has only recently been recognized. CDK5 kinase drives tumor progression and metastasis across various malignancies via different mechanisms in a tissue-specific manner.^60, 61, 62, 63^ Of interest, recent reports suggest involvement of aberrant CDK5 signaling in migration of endothelial cells, lymphangiogenesis and formation of blood vessels.^64, 65^ These findings, taken together, support the hypothesis that TRPV4 overexpression may create a pro-metastatic microenvironment that supports cancer progression by secreting tumor-derived proteins and/or exosomes. It would be interesting to investigate the role of secreted proteins identified in this study, for example, Talin and CDK5 as mediators of TRPV4 metastatic function in future studies.

Finally, we also showed that TRPV4 has a clinical implication on the aggression of breast cancer and survival rate in the breast cancer patients. Our IHC analysis in the clinical samples demonstrated the association of relatively high TRPV4 expression with metastatic lesions, high grade tumor, as well as poorer OS and DFS. Although the mortality due to breast cancer is decreasing because of the early detection, use of hormone therapy and recent introduction of new drugs, management of metastatic and triple negative breast cancer remain challenging. One of the reasons is due to the heterogeneity of the disease. Thus, it is imperative to develop reliable biomarkers and targeted therapeutics for precision medicine. TRPV4 may be an attractive molecule for both purposes as its cell surface nature provide easy access to drug target and is amenable to molecular imaging of metastatic cancer cells. TRPV4 monoclonal antibody-chemo drug conjugate is a possibility for inhibiting disease aggression in patients with high TRPV4 expression in their tumors. However, more in-depth studies will clarify the clinical utility of TRPV4 for breast cancer management.

## Patients and methods

### Patient samples

Tissue microarray (*n*=273) were purchased from Biomax (Derwood, MD 20855, USA)*.* Clinical samples were also obtained from the Singapore General Hospital (SGH; *n*=248) and National University Hospital (NUH; *n*=240). Matched normal and IDC tissues were from SGH (*n*=55) and NUH (*n*=67). Correlation studies on tumor grade (*n*=353), tumor size (*n*=293) and survival analysis (*n*=293) were performed on the samples from SGH and NUH. Institutional Review Board approval was obtained before the study. Samples were randomly acquired to avoid biasness.

### Reagents

4α-alpha-phorbol 12,13-didecanoate, BAPTA-AM and AKT inhibitor IV were from Merck KGaA (Darmstadt, Germany). TRPV4 rabbit polyclonal antibodies were generated in Dr Christian Harteneck’s laboratory using a TRPV4-derived peptide sequence (N terminus-CENPHKKADMRRQDS-C-terminus). The rest of the antibodies were from Cell Signaling Technology (Danvers, MA, USA). Enhanced chemiluminescence detection kit was purchased from General Electric Healthcare, Bio-Sciences (Uppsala, Sweden), prestained molecular weight markers were from Bio-Rad (Hercules, CA, USA), protease inhibitors cocktail were from Roche (Mannheim, Germany) and PolyVinylidene DiFluoride (PVDF) membranes were from Millipore (Bedford, MA, USA). Sodium Orthovanadate and 3% (0.9 M) Hydrogen Peroxide were purchased from Sigma-Aldrich (St Louis, MO, USA).

The Triton-X was purchased from Sigma Chemical (St Louis, MO, USA). Protease & phosphatase inhibitor cocktails were from Roche (Nutley, CA, USA). Tris-base and EDTA were from First Base Laboratories Sdn Bhd (Selangor Darul Ehsan, Malaysia). JetPRIME transfection reagent was supplied by Polyplus Transfection Inc. (New York, NY, USA).

Mouse TRPV4-specific siRNA oligonucleotides were purchased from Invitrogen (Carlsbad, CA, USA) and the siRNA sequences are as following: *Luciferase* GL2: 5′-CGUACG CGGAAUACUUCGA-3′ *Trpv4* siRNA1: 5′-AGAAGCAGCAGGUCGUACAUCUUGG-3′ *Trpv4* siRNA3: 5′-AAACUUGGUGUUCUCUCGGGUGUUG-3′.

### Cell lines

4T07 cell line was obtained from Dr Fred Miller at the Barbara Ann Karmanos Cancer Institute (Detroit, MI, USA) and cultured in Dulbecco's modified Eagle's medium (DMEM) media. TRPV4-HEK293 T-REx cell line was generated in Dr Christian Harteneck’s laboratory, and cultured in DMEM media containing 100 μg/ml Zeocin and 15 μg/ml blasticidin. Both cell lines were supplemented with 10% fetal bovine serum, penicillin (100 U/ml), streptomycin (100 μg/ml) and maintained at 37 °C in a humidified atmosphere of 5% CO_2_.

### Transendothelial migration assay

100000 HUVEC cells were cultured on each 8 μm pore size insert (Cell Biolabs Inc., San Diego, CA, USA) for 48 h. 2 x 10^5^ of the overnight serum starved transfected cancer cells were labeled with fluorescent dye before being added onto the monolayer of the HUVEC cells. The insert was then transferred to a new plate containing fresh medium with 10% fetal bovine serum. Assay was performed at 37 °C for 8 h. Non-migrating cells at the top were removed, whereas cells that have migrated to the bottom of the membrane were first dissociated from the membrane, then lysed and quantified using CyQuant GR fluorescent dye at 480 nm/520 nm. Experiments were performed with three technical replicates and independently validated across two biological experiments.

### Transfection

Cells were seeded at 70–80% confluency in 60 mm dish in medium containing 10% fetal bovine serum and 100 U Penicillin/Streptomycin 1 day before transfection and transfected with 200 nm siRNA and 10 μl jetPRIME reagent (Polyplus Transfection Inc.) according to the manufacturer’s instructions. Non-targeting siRNA duplex pGL2 luciferase or empty-vector served as negative control for knockdown experiment or overexpression experiments, respectively. Cells were harvested 48 h. post-transfection for western blot analysis or used for functional assays.

### Intracellular Ca^2+^ measurement

Cells were loaded with 5 μm fura-2-AM (Molecular Probes) for 30 min at 37 °C in the measuring buffer contained 145 mm NaCl, 5 mm KCl, 1 mm MgCl_2_, 9 mm glucose, 0.2% bovine serum albumin, 10 mm HEPES and 1 mm CaCl_2_ (pH 7.4 with NaOH), dislodged with Trypsin-EDTA before assaying for intracellular calcium concentration in a cuvette under constant, gentle stirring (1 ml final volume). 0.5% Triton-X was added to get R_max_ (as a positive control) and 20 mm EDTA was added to get R_min_ (as a negative control). Fluorescent emission was monitored at 510 nm with alternate excitation at 340 and 380 nm using a RF-5301PC Intracellular Ion Measurement System Spectrofluorophotometer (Super Ion Probe); Shimadzu Corporation. [Ca^2+^]_i_ was calculated based on Grynkiewicz’s two wavelength method.^66^

### Conditioned media preparation

Approximately 80% confluent cells were rinsed thrice with 1 × sterile phosphate-buffered saline and incubated for 24 h in serum-free, protein-free DMEM medium. Following 24 h of starvation, the cells were observed to be healthy and subsequently the media containing the secreted proteins were collected. The collected media was filtered using a 0.22 μm filter (Millipore, Bedford, MA, USA) to remove contaminants from floating cells as well as dead cells and cell debris present in the culture media. Subsequently, the filtered media was concentrated using Amicon Ultra-15 and Ultra-4 Centrifugal Filter Units (Millipore, Billerica, MA). Bradford assay, with bovine serum albumin as the protein standard, was then used to determine the concentration of secreted proteins (Thermo Scientific, Waltham, MA, USA).

### iTRAQ-LC/MS/MS-based identification and relative quantification of secreted proteins

Secreted proteins collected from each cell line were subjected to iTRAQ labeling according to manufacturer’s protocol (Applied Biosystems, Framingham, MA, USA). In brief, 200 μg of secreted proteins were reduced and cysteine blocked before digestion overnight with trypsin. Following iTRAQ labeling, the peptides were dissolved in Buffer A which contained 5 mm KH_2_PO_4_ and 25% ACN (pH 2.7). Fractionation of peptides was performed using 1260 Infinity High Performance Liquid Chromatography (Agilent Technologies, Santa Clara, CA, USA), using a PolySULFOETHYL A column, 200 × 4.6-mm, 5 μm, 200-Å (PolyLC Inc., Columbia, MD, USA). A 60 min step gradient was used where the gradient started with 100% of Buffer A for 5 min, followed by a ramp from 5 to 21% of Buffer B (5 mM KH_2_PO_4_, 25% ACN, 500 mM KCl, pH 2.7) for 35 min, then ramping from 30 to 100% of Buffer B for 15 min and maintained at 100% of Buffer B for 5 min. The fractions obtained were lyophilized in a vacuum concentrator and subsequently cleaned via a C-18 cleanup using C18 Discovery DSC-18 SPE column (100 mg capacity, Supelco, Sigma-Aldrich). The cleaned fractions were again lyophilized and then stored at −20 °c before mass spectrometric analysis.

Each cleaned peptide fraction was dissolved in 35 μl of Buffer A which contained 2% ACN in 0.1% formic acid. Each sample of 10 μl was injected into the nano-LC-ESI-MS/MS system by an autosampler for each analysis. Mass spectrometry was performed by QStar Elite Hybrid ESI Quadrupole time of flight tandem mass spectrometer (Applied Biosystems, Framingham, MA, USA) coupled to an online capillary liquid chromatography system (Tempo nLC, Applied Biosystems). The column used to separate the peptide mixture was a PepMap C-18 RP capillary column (Dionex, The Netherlands). Peptide mixture separation was conducted at 0.3 μl/min, on a 125 min gradient. The gradient started with 4% of Buffer B (98% ACN in 0.1% formic acid) and 96% of Buffer A for 3 min. Two ramping gradients of 4% of Buffer B in 7 min, 10–35% of Buffer B for 55 min and 35–100% of Buffer B for 25 min followed by a hold at 100% of Buffer B for 15 min and 96% of Buffer A for 20 min. The mass spectrometer was set in the positive ion mode, with a selected mass range of 300–1800 *m*/z. The time of summation of MS/MS events was adjusted to 2 s. Two charged peptides with the highest abundance above a 20 count threshold were chosen for MS/MS and dynamically excluded for 30 s with a mass tolerance within the range of ±50 mDa.

Protein identification and quantification for iTRAQ samples were carried out by importing the data files (*.wiff) into ProteinPilot software and analyzed using the Paragon algorithm (version 2.0; Applied Biosystems, MDS-Sciex). The search was performed against the International Protein Index human (version 3.87, date of release: September 2011). The search parameters allowed cysteine modification of MMTS and biological modifications pre-defined in the software. The detected protein threshold (unused protscore (conf) in the software was set to 1.3 to achieve 95% confidence. iTRAQ ratios are calculated based on the relative cluster areas of the iTRAQ reporter fragment peaks 115 (Luc_DMSO), 116 (S1_DMSO), 117 (S3_DMSO), 118 (Luc_PDD), 119 (S1_PDD) and 121 (S3_PDD). To estimate the rate of false-positive in the data set obtained, a database search against a concatenated pseudo reverse database was employed.^67^ Using this strategy, the false-positive discovery rate for this data set is estimated to be 1.02%. The false-positive discovery rate is defined to be the percentage of decoy proteins identified against the total protein identification.

### Immunohistochemistry

Tissue microarray sections of 4-μm thickness were dewaxed in xylene, followed by graded alcohol (100, 95 and 70%) and water. Heat-induced epitope retrieval was done by heating slides in Cell Conditional Solution pH 9.0 (Ventana Medical Systems) at 100 °C for 60 min. Immunostaining was performed using Ventana Autostainer. After blocking endogenous peroxidase activity for 4 mins, tissues sections were incubated with primary rabbit polyclonal antibody against TRPV4 (1:200 dilution) for 30 min at 37 °C. Detection was done using the Ultraview Universal DAB detection kit (Ventana #760-500) according to the manufacturer’s instructions. The sections were then counterstained with haematoxylin, dehydrated and mounted in depex.

All IHC results were reviewed by certified pathologist. Staining was scored by intensity −0 (no staining), 1+ (mild), 2+ (moderate) and 3+ (strong). IHC score of more than 1.0 was defined as ‘TRPV4 high’. Comparisons between groups were performed using Wilcoxon Signed rank test or Matt–Whitney *U* test. Correlation between TRPV4 IHC scores and clinicopathological parameters were determined using chi square or fisher exact test. OS and DFS curves were constructed using Kaplan–Meier method and the survival curves were compared using the Log-rank test. DFS and OS were defined as time from date of diagnosis to date of first recurrence and death or last follow-up date, respectively. All analyses were performed using SPSS version 20. *P***-**values <0.05 were considered to be statistically significant *P<0.05, **P<0.01, ***P<0.001.

### Statistical Methods

Cell-based assays and western blotting were analyzed by unpaired two-tailed Student’s *t*-test.

## Figures and Tables

**Table 1:** Extracellular proteins whose expressions were regulated by 4α-PDD in a TRPV4-specific manner, that is, proteins that showed PDD-induced changes in control siRNA transfected but not in TRPV4-knockeddown cells

	*Mode of secretion*	*Fold change*	*MW (kDa)*	*% Coverage*	P*-value*
*TRPV4-specific proteins downregulated following PDD treatment*					
Gene_Symbol=Spp1 Osteopontin	Classical	0.68	36	76.19	3.26E−14
Gene_Symbol=Mtpn Myotrophin	Unconfirmed	0.75	14	49.15	1.17E−02
Gene_Symbol=B2m Beta-2-microglobulin	Classical	0.71	14	60.50	4.00E−06
Gene_Symbol=Clu Clusterin	Classical	0.70	54	76.34	1.47E−04
Gene_Symbol=Tinagl1 Tubulointerstitial nephritis antigen-like	Classical	0.73	56	26.39	1.71E−02
Gene_Symbol=E330026B02Rik Isoform 1 of Collagen alpha-6(VI) chain	Classical	0.74	142	18.94	4.97E−02
Gene_Symbol=Fn1 Putative uncharacterized protein	Classical	0.77	297	54.34	3.36E−02
Gene_Symbol=Atp5b ATP synthase subunit beta, mitochondrial	Exosome	0.73	63	53.50	3.23E−02
Gene_Symbol=Igfbp4 Insulin-like growth factor-binding protein 4	Classical	0.74	30	66.93	4.82E−02
Gene_Symbol=Psap Sulfated glycoprotein 1	Unconfirmed	0.73	67	33.75	5.18E−03
Gene_Symbol=Filip1 Filamin A interacting protein 1	Unconfirmed	0.61	138	32.21	9.42E−03

*TRPV4-specific proteins upregulated following PDD treatment mode of secretion*					
Gene_Symbol=Gm1821 Putative uncharacterized protein	Unconfirmed	2.12	18	99.35	6.37E−07
Gene_Symbol=Dbi Acyl-CoA-binding protein	Unconfirmed	1.36	10	73.56	2.17E−06
Gene_Symbol=Nme2 Nucleoside diphosphate kinase B	Exosome	1.34	18	96.05	1.24E−02
Gene_Symbol=Psme1 Proteasome activator complex subunit 1	Exosome	1.43	30	57.03	1.85E−02
Gene_Symbol=Asns Asparagine synthetase [glutamine-hydrolyzing]	Exosome	1.42	67	31.55	2.49E−02
Gene_Symbol=ENSMUSG00000072940; Rps28 40 S ribosomal protein S28	Unconfirmed	1.38	8	52.17	3.40E−02
Gene_Symbol=- Ring-finger protein 213	Unconfirmed	1.32	591	35.64	3.52E−01
Gene_Symbol=Sparcl1 SPARC-like protein 1	Classical	1.35	78	16.46	2.61E−02
Gene_Symbol=Tsen34 34 kDa protein	Unconfirmed	1.42	38	74.36	1.26E−02
Gene_Symbol=Tln1 Talin-1	Exosome	2.02	270	35.22	5.78E−05
Gene_Symbol=Tubb2c Tubulin beta-2C chain	Unconfirmed	1.60	53	60.45	4.91E−02
Gene_Symbol=Hdgf 22 kDa protein	Classical	1.34	22	24.26	1.69E−03
Gene_Symbol=Lrpprc Leucine-rich PPR motif-containing protein	Exosome	1.39	130	35.34	2.41E−02
Gene_Symbol=Ywhae 14-3-3 protein epsilon	Exosome	1.31	31	80.39	3.19E−02
Gene_Symbol=Trim28 Isoform 2 of Transcription intermediary factor 1-beta	Unconfirmed	1.40	100	67.00	2.68E−02
Gene_Symbol=Tagln2 Transgelin-2	Exosome	1.31	22	85.43	6.46E−03

Abbreviations: 4α-PDD, 4α-phorbol 12,13-didecanoate; siRNA, small interfering RNA.

**Table 2:** Gene Ontology classifying TRPV4 target proteins into their molecular functional groups and biological processes using Metacore analysis

*#*	*Networks*	*Genes from active data*	*Total*	P*-value*
1	Cytoskeleton-regulation of cytoskeleton rearrangement	β-Tubulin, 14-3-3 epsilon, Talin, 14-3-3	183	9.62E−04
2	Proteolysis-ECM remodeling	Fibronection, Clusterin and Osteopontin	85	1.17E−03
3	Cell adhesion-integrin-mediated cell-matrix adhesion	Fironection, β-Tubulin, Talin and Osteopontin	214	1.72E−03

The top 3 functional networks most significantly associated with these 27 candidates are shown.

**Table 3:** Extracellular proteins whose expression were significantly affected by *Trpv4*-gene silencing alone

*TRPV4-specific protein expression affected by Trpv4-gene silencing*	*Mode of secretion*	*MW (kDa)*	*% Coverage*	*Fold change S1_DMSO: Luc_DMSO*	P*-value*	*Fold change S3_DMSO: Luc_DMSO*	P*-value*
Gene_Symbol=Cdk5 Cell division protein kinase 5	Exosome	33	45.55	0.59	5.17E−16	0.72	1.09E−14
Gene_Symbol=Ube2l3 Ubiquitin-conjugating enzyme E2 L3	Exosome	17	42.86	0.71	0.01	0.64	2.56E−4.0
Gene_Symbol=Pcf11 Cleavage and polyadenylation factor subunit homolog	Exosome	173	27.88	1.67	0.01	1.40	4.28E−5.0
Gene_Symbol=Nefm Neurofilament medium polypeptide	Exosome	160	44.10	1.39	0.03	1.30	4.09E−3.0
