# Supplementary material for: TRPV4 plays a role in breast cancer cell migration via Ca2+-dependent activation of AKT and downregulation of E-cadherin cell cortex protein
Source: Oncogenesis. 2017 May 22;6(5):e338–. doi: 10.1038/oncsis.2017.39 (PMC5523072; doi:10.1038/oncsis.2017.39)
Supplement: Supplementary Information [file oncsis201739x1.docx]

**TRPV4 plays a role in breast cancer cell migration via Ca^2+^ dependent activation of AKT and down-regulation of E-cadherin cell cortex protein**

Wen Hsin Lee^1†^, Lee Yee Choong^1†^, Tan Hock Jin^1^, Naing Naing Mon^1^, Shirly Chong^1^, Chiat Siang Liew^1^, Thomas Putti^2^, SsuYi Lu^1^, Christian Harteneck^3^ and Yoon Pin Lim^1,4,5*^

*^1^Department of Biochemistry, Yong Loo Lin School of Medicine, National University of Singapore;^2^Department of Pathology, National University of Singapore and National University Hospital; ^3^Department of Pharmacology and Experimental Therapy, Institute of Experimental and Clinical Pharmacology and Toxicology, Eberhard Karls University Hospitals and Clinics, Tübingen, Germany; ^4^NUS Graduate School for Integrative Sciences and Engineering, Singapore; ^5^National University Cancer Institute, National University Health System.*

*† The authors contributed equally*

* To whom all correspondence should be addressed: Yoon Pin LIM, Department of Biochemistry, MD4 Level 1, 5 Science Drive 2, Singapore 117545. Tel: (65) 66011891; Fax: (65) 68739664.

E-mail: bchlyp@nus.edu.sg

*Running Title: TRPV4-mediated regulation of AKT and E-cadherin*

**Keywords**: TRPV4, AKT, E-cadherin, breast cancer, calcium signaling

**Supplementary Figure 1** – IHC analysis confirmed the specificity of anti-TRPV4 antibodies used in the IHC analysis through peptide competition studies. Cep68 peptide served as a non-specific peptide.

**Supplementary Figure 2** – shows activation of AKT, pFAK and down-regulation of E-cadherin, beta-catenin by PDD treatment in 4T07 cells.

**Supplementary Figure 3** – Transendothelial migration assay shows AKT is required for TRPV4-mediated migration. 4T07 cells were treated with AKT inhibitor (5uM) and/or arachidonic acid which (AA, 20 uM) in serum-free media over 8 hr of time course prior to analysis. Complete growth media was used as chemoattractant in the lower chamber of the transwell plate. As for co-treatment, cells were first pre-treated with AKT inhibitor for 1 hr. (n=12, data points represent mean ±SEM).

**Supplementary Figure 4** – HeLa cells were transfected with empty vector or TRPV4-expresing plasmid for 48 hours before PDD treatment at indicated time point.
